# Non-Small Cell Lung Cancer Cells Expressing CD44 Are Enriched for Stem Cell-Like Properties

**DOI:** 10.1371/journal.pone.0014062

**Published:** 2010-11-19

**Authors:** Elaine Lai-Han Leung, Ronald R. Fiscus, James W. Tung, Vicky Pui-Chi Tin, Lik Cheung Cheng, Alan Dart-Loon Sihoe, Louis M. Fink, Yupo Ma, Maria Pik Wong

**Affiliations:** 1 Department of Pathology, The University of Hong Kong, Hong Kong, Special Administrative Region, China; 2 Cancer Molecular Biology Section, Nevada Cancer Institute, Las Vegas, Nevada, United States of America; 3 College of Pharmacy, University of Southern Nevada, Henderson, Nevada, United States of America; 4 Division of Laboratory Medicine, Nevada Cancer Institute, Las Vegas, Nevada, United States of America; 5 Cardiothoracic Surgery Unit, Queen Mary Hospital, Hong Kong, Special Administrative Region, China; 6 Department of Pathology, Stony Brook University Medical Center, Stony Brook, New York, United States of America; University of Hong Kong, China

## Abstract

**Background:**

The cancer stem cell theory hypothesizes that cancers are perpetuated by cancer stem cells (CSC) or tumor initiating cells (TIC) possessing self-renewal and other stem cell-like properties while differentiated non-stem/initiating cells have a finite life span. To investigate whether the hypothesis is applicable to lung cancer, identification of lung CSC and demonstration of these capacities is essential.

**Methodology/Principal Finding:**

The expression profiles of five stem cell markers (CD34, CD44, CD133, BMI1 and OCT4) were screened by flow cytometry in 10 lung cancer cell lines. CD44 was further investigated by testing for *in vitro* and *in vivo* tumorigenecity. Formation of spheroid bodies and *in vivo* tumor initiation ability were demonstrated in CD44^+^ cells of 4 cell lines. Serial *in vivo* tumor transplantability in nude mice was demonstrated using H1299 cell line. The primary xenografts initiated from CD44^+^ cells consisted of mixed CD44^+^ and CD44^−^ cells in similar ratio as the parental H1299 cell line, supporting *in vivo* differentiation. Semi-quantitative Real-Time PCR (RT-PCR) showed that both freshly sorted CD44^+^ and CD44^+^ cells derived from CD44^+^-initiated tumors expressed the pluripotency genes *OCT4/POU5F1*, *NANOG*, *SOX2*. These stemness markers were not expressed by CD44^−^ cells. Furthermore, freshly sorted CD44^+^ cells were more resistant to cisplatin treatment with lower apoptosis levels than CD44^−^ cells. Immunohistochemical analysis of 141 resected non-small cell lung cancers showed tumor cell expression of CD44 in 50.4% of tumors while no CD34, and CD133 expression was observed in tumor cells. CD44 expression was associated with squamous cell carcinoma but unexpectedly, a longer survival was observed in CD44-expressing adenocarcinomas.

**Conclusion/Significance:**

Overall, our results demonstrated that stem cell-like properties are enriched in CD44-expressing subpopulations of some lung cancer cell lines. Further investigation is required to clarify the role of CD44 in tumor cell renewal and cancer propagation in the *in vivo* environment.

## Introduction

Lung cancer is the leading cause of cancer deaths worldwide. The overall prognosis is poor with low 5 year survival due to late presentation, disease relapse and lack of curative systemic therapy. Recently, the cancer stem cells (CSC) theory proposes that cancers are maintained by subpopulations of tumor cells that possess stem or progenitor cell characteristics. These cells can initiate tumor formation, differentiate along multi-potent pathways and are relatively resistant to conventional chemotherapy [1]. CSC have been demonstrated in haematological and some solid tumors such as breast, brain, colon, lung and liver cancers [2], [3], [4], [5], [6]. Various stem cell markers of normal tissues have been used for CSC identification and isolation. For example, CD133 is the most frequently demonstrated marker in cancers of the liver, brain, colon and lung, etc [3], [4], [5], [6]. The CD44^+^/CD24^−/low^ profile characterizes CSC in breast and prostate cancers [7], [8]. Expression of the key ‘stemness’ genes of embryonic (ES) and induced-pluripotent stem (iPS) cells OCT4 and BMI1 [9], [10] have been found in CSC from different cancers [11], [12].

The marker profile of lung CSC remains to be explored. Recent studies using NSCLC cell lines and fresh lung tumor tissues suggest CD133 as the lung CSC marker [3], [11], [13], [14], [15], [16]. Biochemical studies showed that CD133 plays a functional role in cell cycle regulation and proliferation but not tumor initiation [17]. Studies in colon cancer showed that CD133^+^ cells have a higher DNA content [18] and become CD133^−^ during metastasis, but both CD133^+^ and CD133^−^ cells initiate tumor in SCID mice [19]. CD133^−^ populations from colon cancer and melanoma were also found to be tumorigenic in SCID/nude mice [19], [20]. Markers such as ESA, CXCR4, ALDH and ABCG2 have been used with CD133 for isolating CSC from lung cancers [13], [21], [22]. CD34 and Sca-1 are useful for identifying murine lung stem cells but Sca-1 is not expressed in human tissues [23]. To explore further lung CSC markers, we have screened the expression profile of CD34, CD44, CD133, BMI1 and OCT4 in 10 lung cancer cell lines by flow cytometry. We demonstrated that CD44^+^ but not CD44^−^ cells from selective cancer cell lines could be expanded and serially propagated *in vitro* and *in vivo*. We propose that CD44-expressing cells are enriched for stem cell properties. Our study does not show that all CD44^+^ cells are bona fide CSC, however, we propose that CD44 could be a marker of tumor initiation ability in some lung cancer cells. The expression pattern of CD44 was also characterized in clinical lung cancers.

## Results

### Stem cell marker expression profile of NSCLC cell lines

**Figure 1:**
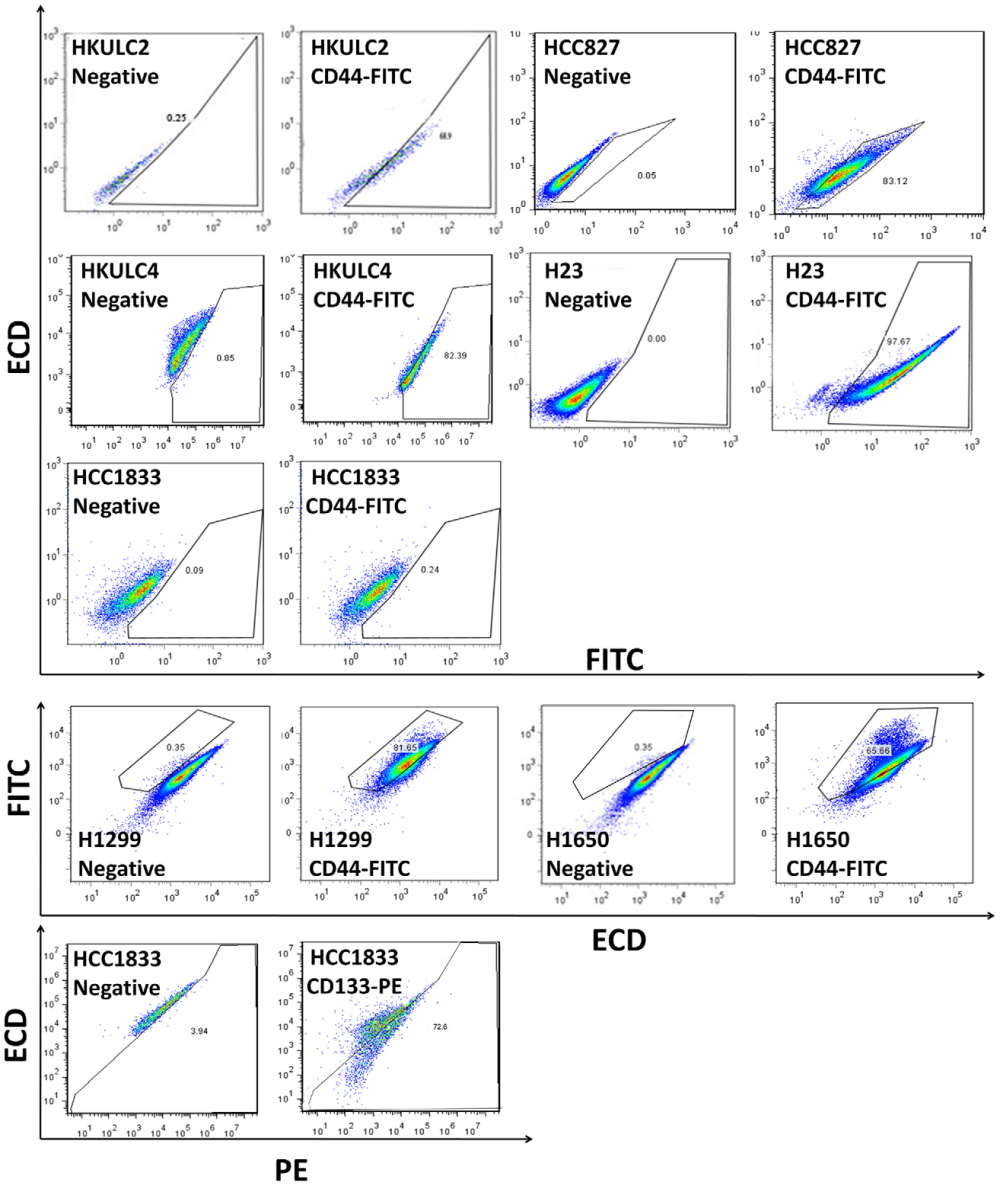
Surface marker expression profile. Representative diagrams of flow cytometry analysis of CD44 (FITC) and CD133 (PE) expression in lung cancer cell lines HKULC2, HCC827, HKULC4, H23, HCC1833, H1299 and H1650. Dead cells, cell debris and doublets were gated out. Compensation for background fluorescence was performed by measuring target signals of single color controls and negative controls. Data were presented in 2D diagrams plotting either PE or FITC signals against an irrelevant channel ECD® (also known as PE-Texas Red). The average percentages from 3 individual analyses were presented in Table 1.

**Figure 2:**
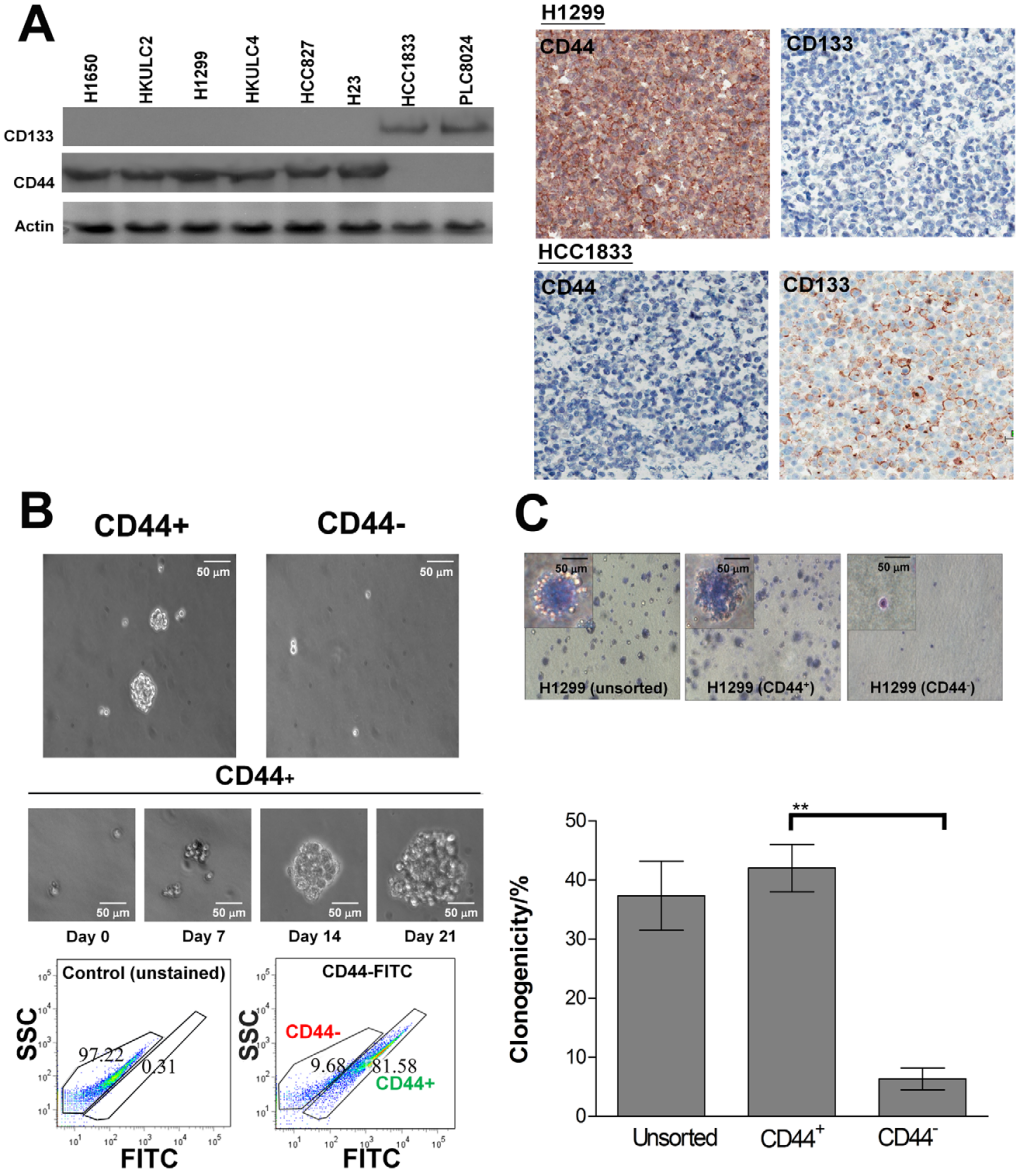
*In vitro* tumorigenecity of NSCLC cells. A. Representative immunoblot of H1650, HKULC2, H1299, HKULC4, HCC827, H23, HCC1833 and PLC8024 total protein lysate using antibodies against CD44 and CD133. Actin was used as a loading control. The blot was representative of three individual experiments. B. Overall view to show density of SB formation from CD44^+^ and CD44^−^ H1299 cells after culturing in RPMI medium with EGF, FGF and insulin supplement for 21 days (upper panel), and morphology of SB at weekly intervals (middle panel). Cells of the first generation SB were disaggregated and analyzed by flow cytometry (lower panel). The CD44^+^ to CD44^−^ (81.58% to 9.68%) ratio was similar to that of the parental H1299 cells (81.3% to 18.7%, Table 1). SSC, Side Scatter Channel. C. Representative fields of colony formation of unsorted, CD44^+^ and CD44^−^ H1299 cells in soft-agar after culturing for 21 days. Insets show magnified views of representative colonies from the respective cells taken under the same magnification. CD44^−^ subgroup showed significantly fewer colonies compared to both CD44^+^ and unsorted subgroups. (**, p<0.01). Histograms represented average of 3 independent observations from separate experiments.

We analyzed the expression profile of putative surface (CD34, CD44, CD133) and nuclear markers (BMI1, OCT4) of stem cells in 10 lung cancer cell lines using flow cytometry (Table 1). Of the surface markers, CD34, CD44 and CD133 were expressed at various frequencies. CD44 was the major marker expressed by H1299 and H23. A549, H441 and H1648 showed no expression of the surface markers studied. CD133 was expressed in HCC1833 only. No correlation in expression frequency was observed between any 2 markers. Both the nuclear markers, BMI1 and OCT4, were expressed in the majority of cancer cells in all cell lines studied. Representative diagrams of flow cytometry analysis for CD44 and CD133 expression were shown in Fig 1. Immunoblotting and immunohistochemistry (IHC) analyses were also performed on cell lines that contained CD44^+^ and CD133^+^ populations (Fig 2A). Immunoblot showed CD44 protein expression in H1650, HKULC2, H1299, HKULC4, HCC827 and H23. CD133 protein was only expressed in HCC1833 and PLC8024 which was used as a positive control cell line for CD133 [4]. IHC also showed CD133 expression in HCC1833 but not H1299, and vice versa for CD44 expression. Thus, results of both analyses were in line with flow cytometry data.

**Table 1:** Expression of Putative Cancer Stem Cell Markers in NSCLC Cell Lines.

	Cell lines	CD34	CD44	CD133	BMI1	OCT4
1	H1650	2.9	61.7	0.0	99.6	70.7
2	HKULC2*	10.3	68.6	0.0	92.3	98.3
3	H1299	0.0	81.3	0.0	89.1	73.8
4	HKULC4*	7.8	82.4	0.0	94.3	87.6
5	HCC827	9.2	82.5	0.0	98.1	88.3
6	H23	0.1	95.9	0.0	99.9	91.0
7	HCC1833	2.3	0.0	80.0	97.4	99.1
8	A549	0.0	0.0	0.0	97.5	97.0
9	H441	0.0	0.0	0.0	99.9	99.9
10	H1648	0.0	0.0	0.0	99.1	99.7

*Cell lines raised from local patients (46).

Results represented average of three individual flow cytometry experiments.

### Spheroid formation ability of NSCLC cell lines

We next assessed spheroid body (SB) formation ability of FASC-fractionated cells according to CD44 and CD133 expression in 7 cell lines. 500 unsorted, marker^+^ and marker^−^ cells, respectively, were cultured in non-adhesive and serum-free conditions with EGF, bFGF and insulin supplements for 21 days. The percentage of SB formation of each cell line was counted by random field selection at day 21 and results were plotted as histograms (Supplementary Figure S1). Unsorted cells from all 7 cell lines were able to form SB. SB were also formed from CD44^+^ subpopulations of 6 cell lines in which CD44 was expressed, but not from the CD44^−^ subpopulations. In addition, HKULC2, H1299 and H1650 which contained lower initial proportions of CD44^+^ cells gave rise to significantly more SB compared to unsorted cells (*p<0.05, **p<0.01). For HCC1833, CD133^+^ but not CD133^−^ cells formed SB. The increase in SB numbers from CD133^+^ compared to unsorted cells was not statistically significant. After 21 days, all SB from H1299 CD44^+^ cells were dissociated into single cells and re-suspended in culture media. Up to three serial passages were established, indicating *in vitro* self-renewal of the CD44^+^ cells. Flow cytometry analysis of the first generation SB cells from H1299 showed 81.58% of CD44^+^ cells, which closely resembled the 81.3% of the parental cells, demonstrating that *in vitro* tumorigenecity from CD44^+^ cells resulted in a progeny with the same profile of CD44 expression (Fig. 2B). Clonogenicity assay in soft agar also showed that freshly isolated H1299 CD44^+^ cells formed significantly more colonies than CD44^−^ cells in 3 weeks (**p<0.01), supporting enhanced self-renewal capacity of CD44-selected cells (Fig. 2C).

### *In vivo* tumor-initiating properties of CD44^+^ cells and sequential increase of transplantation efficiency in nude mice

**Figure 3:**
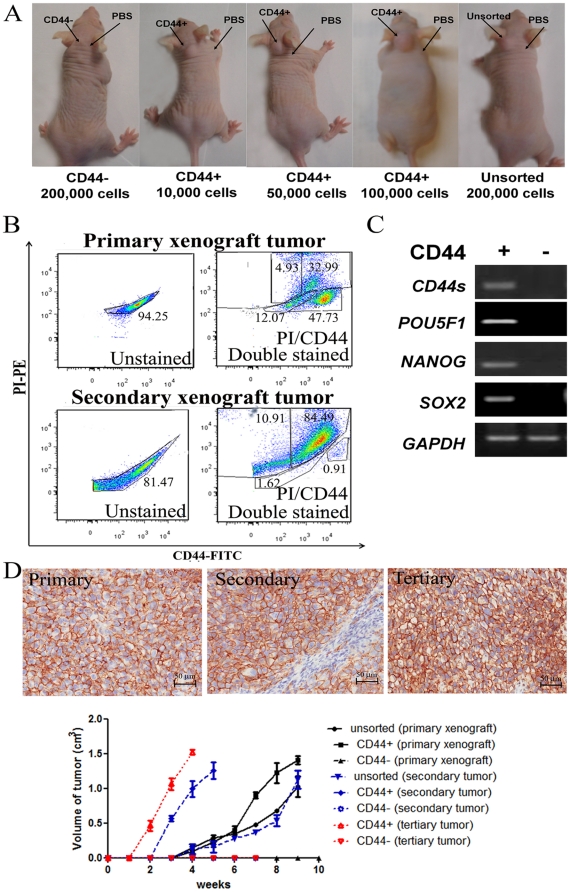
*In vivo* tumorigenicity assay of H1299 cells. A. Representative pictures of primary xenograft tumors. Tumor initiation was achieved by 200,000 unsorted cells (3/4 mice). From CD44^+^ cells, tumors were initiated by 10,000 (1/4 mice), 50,000 (3/4 mice) and 100,000 cells (4/4 mice). No tumor was formed with 200,000 CD44^−^ cells (0/4 mice). PBS, saline injection control. B. Flow cytometry analysis of disaggregated primary and secondary xenografts showed similar CD44^+^ to CD44^−^ subgroup ratios as parental H1299 cells. X-axis: CD44 expression, FITC channel; Y-axis: Dead cells stained by PI, PE channel. C. Semi-quantitative RT-PCR analysis of primary xenografts showed expression of CD44 standard form (CD44s) and pluripotency genes (*POU5F1*, *NANOG*, *SOX2*) in CD44^+^ but not CD44^−^ subgroups. D. IHC analysis of xenografts from sequential generations of recipient mice showed CD44 expression in cell membrane distribution. Host cells of mouse origin present in the tumor stroma were not stained. Tumor growth curves of the primary, secondary and tertiary xenograft tumors formed by unsorted and CD44-sorted H1299 cells were plotted by observing and measuring tumor formation weekly for up to 9 weeks, afterwhich mice were scarified to avoid overgrowth of tumors.

The ability of the marker-selected cells to initiate *in vivo* tumor was investigated by subcutaneous transplantation into nude mice. For H1299, HKULC4, H1650 and HCC827, as few as 10,000 CD44^+^ cells were able to initiate tumors in 30–68 days (Table 2), but no tumor was formed from the same number of unsorted or CD44^−^ cells after 90 days. For unsorted cells of H1299, tumor initiation could be achieved by 200,000 cells (3/4 mice), while with CD44+ cells, tumors were initiated by 10,000 (1/4 mice), 50,000 (3/4 mice) and 100,000 cells (4/4 mice). For CD44^−^ cells, no tumor was formed even using 200,000 cells (0/4 mice) (Fig. 3A-representative tumors, Table 2). We have dissected the mice and examined all the organs and found no metastatic tumor formation from the sorted or unsorted cells under the observed period. For HKULC2 and H23, although SB formation was observed, no xenograft tumor was formed using 200,000 unsorted, CD44^+^ or CD44^−^ cells. Likewise, 200,000 unsorted, CD133^+^ or CD133^−^ HCC1833 cells did not form tumors.

**Table 2:** *In Vivo* Tumorigenecity of Marker^+/−^ NSCLC cells.

Cell type	Cell number injected	Tumor incidence	Latency (days)
**H1299 unsorted**	10,000	0/4	N/A
	50,000	0/4	N/A
	200,000	3/4	30
**H1299 CD44−**	10,000	0/4	N/A
	50,000	0/4	N/A
	200,000	0/4	N/A
**H1299 CD44+**	10,000	1/4	30
	50,000	3/4	30
	100,000	4/4	30
**HKULC4 unsorted**	10,000	0/4	N/A
	200,000	4/4	35
**HKULC4 CD44−**	10,000	0/4	N/A
**HKULC4 CD44+**	10,000	1/4	35
**H1650 unsorted**	10,000	0/4	N/A
	200,000	4/4	60
**H1650 CD44−**	10,000	0/4	N/A
**H1650 CD44+**	10,000	2/4	60
**HCC827 unsorted**	10,000	0/4	N/A
	200,000	4/4	68
**HCC827 CD44−**	10,000	0/4	N/A
**HCC827 CD44+**	10,000	3/4	68
**HKULC2 unsorted**	10,000	0/4	N/A
	200,000	0/4	N/A
**HKULC2 CD44−**	10,000	0/4	N/A
	200,000	0/4	N/A
**HKULC2 CD44+**	10,000	0/4	N/A
	200,000	0/4	N/A
**H23 unsorted**	10,000	0/4	N/A
	200,000	0/4	N/A
**H23 CD44−**	10,000	0/4	N/A
	200,000	0/4	N/A
**H23 CD44+**	10,000	0/4	N/A
	200,000	0/4	N/A
**HCC1833 unsorted**	10,000	0/4	N/A
	200,000	0/4	N/A
**HCC1833 CD133−**	10,000	0/4	N/A
	200,000	0/4	N/A
**HCC1833 CD133+**	10,000	0/4	N/A
	200,000	0/4	N/A

N/A: results not available.

The next experiments tested whether the tumor initiation capacity could be propagated *in vivo*. Since H1299 CD44+ showed the shortest latency of tumor formation (Table 2), we dissociated H1299 primary tumor to demonstrate *in vivo* serial tranplantability. Only viable tumor cells from disaggregated H1299 xenografts were selected for serial tumor transplantation into secondary, and subsequently, tertiary recipient mice. Tumors were formed from CD44^+^ but not CD44^−^ cells (Table 3). Notably, although the proportions of live CD44^+^ cells (47.73% for primary, 0.91% for secondary) (Fig 3B) were reduced on serial transplantation, the efficiency of tumor initiation increased in successive generations of tumor. The latency of tumor formation from 5,000 CD44^+^ cells was progressively shortened from 30 days of the first generation, to 21 days of the second and 14 days of the tertiary generation, respectively. The number of cells required to initiate tertiary tumors also decreased to 1,000 CD44^+^ cells.

**Table 3:** *In vivo* Serial Transplantation Experiments of CD44^+^ and CD44^−^ Cells Sorted from H1299 Xenograft Tumor.

Cell type	Cell number injected	Secondary tumor incidence	Latency (days)	Tertiary tumor incidence	Latency (days)
**unsorted**	200,000	3/4	28		
**CD44+**	500	0/4	N/A	0/4	N/A
	1,000	0/4	N/A	2/4	14
	5,000	2/4	21	3/4	14
	10,000	2/4	21		
	50,000	3/4	14		
**CD44−**	1,000			0/4	N/A
	5,000			0/4	N/A
	50,000	0/4	N/A		
	100,000	0/4	N/A		

N/A: results not available; blank: tests not done or number of cells unavailable.

To analyze the differentiation capacity of CD44^+^ cells, the harvested tumors were disaggregated and subjected to flow cytometry analysis. The CD44^+^∶CD44^−^ ratio of the primary tumor was 80.72∶17.0, and of the secondary tumor was 85.4∶12.53. These were in the same range as the parental cells (81.3∶ 18.7), demonstrating similar CD44 expression hierarchy of sequential generations of xenografts (Fig. 3B). Further analysis of the primary tumor by RT-PCR revealed the expression of the pluripotency markers *POU5F1*, *NANOG* and *SOX2* in CD44^+^ but not CD44^−^ subpopulations (Fig. 3C).

Fig. 3D shows representative IHC staining for CD44 and tumor growth curves of the primary and serially transplanted xenografts. As shown by the tumor growth curve, the primary and serially transplanted tumors initiated from 50,000 CD44^+^ cells grew faster than 200,000 unsorted cells.

### CD44^+^ cells expressed pluripotency and Epithelial-Mesenchymal-Transition (EMT) markers and possessed differentiation potentials

**Figure 4:**
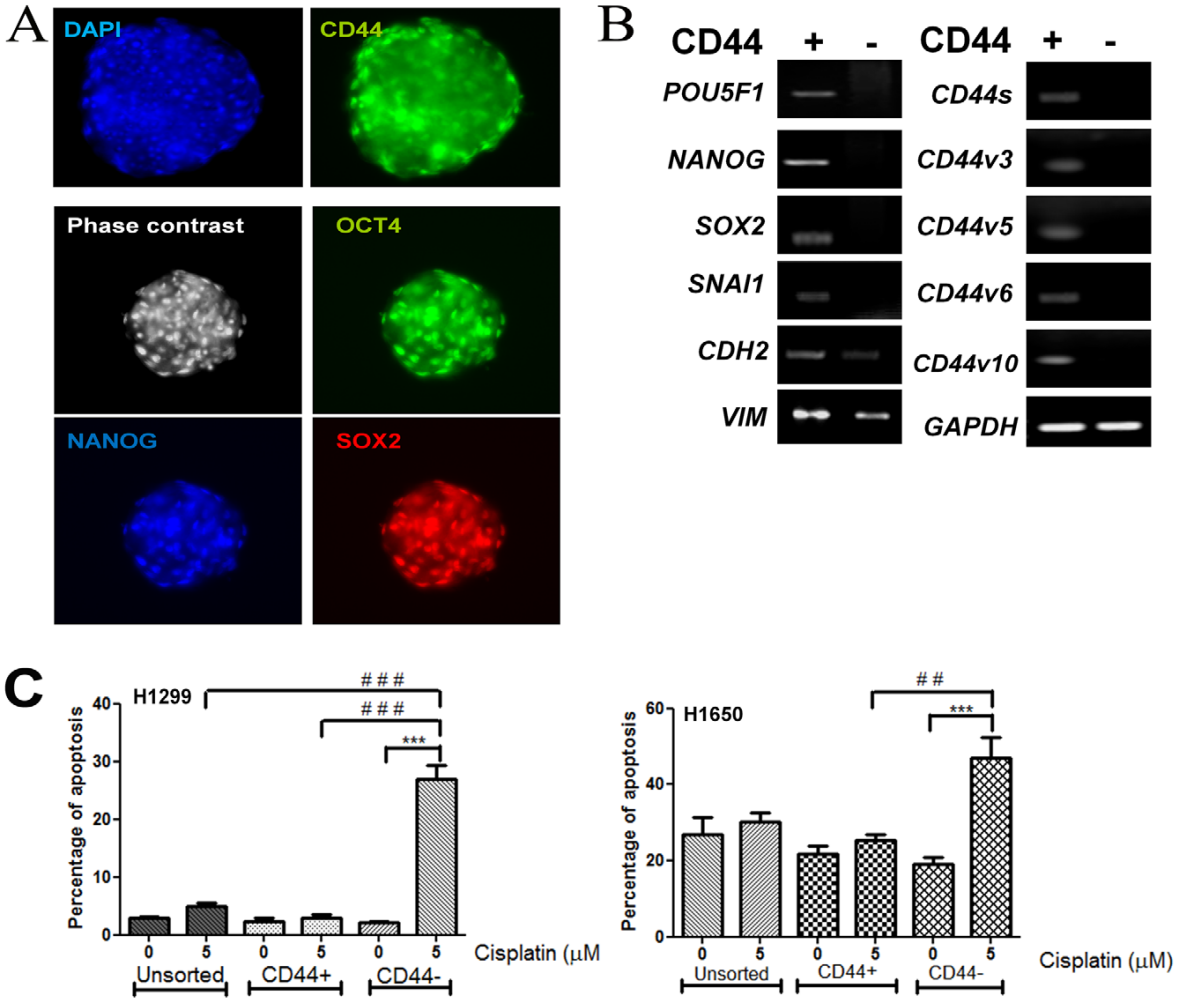
Functional characterization of CD44-selected H1299 cells. A. Immunofluorescence characterization of SB from CD44^+^ subpopulation, showing co-expression of OCT4, NANOG and SOX2. DAPI, control; Green, OCT4-FITC; Red, SOX2-Texas Red; Blue, pseudocolor of NANOG by PE-conjugated antibody. B. Semi-quantitative RT-PCR analysis showed pluripotency genes, *SNAI1*, *CDH2*, *VIM*, *CD44s* and *CD44 v3,5,6,10* expression in CD44^+^ cells. CD44^−^ H1299 cells lack these expression except for *CDH2* and *VIM*. C. CD44^+^ cells were more resistant to the apoptotic effects of 5 µM cisplatin compared to CD44^−^ in H1299 and H1650 cells after 24 hr incubation. Histograms represented average of three individual experiments of unsorted, CD44+ and CD44− cells before and after cisplatin treatment.

The expression of pluripotency genes and acquisition of mesenchymal traits have been shown to indicate a stem cell phenotype. We demonstrated co-expression of the embryonal proteins OCT4, NANOG and SOX2 by IF studies on SB from CD44^+^ H1299 cells. Semi-quantitative RT-PCR was also performed on freshly sorted CD44^+^ and CD44^−^ H1299 cells. Expression of *OCT4*, *NANOG*, *SOX2* and the mesenchymal markers *SNAI1*, *CDH2* and *VIM* were shown in CD44^+^ cells but were lower or undetectable in CD44^−^ cells. Since differential expression of the standard (*s*) and variant forms (*v3*, *v5*, *v6* and *v10*) of CD44 has been demonstrated in stem or differentiated cells, we investigated the pattern of *CD44* mRNA splicing by RT-PCR. Both the standard and variant forms were found in CD44^+^ but not CD44^−^ cells, suggesting retention of *CD44* mRNA differential splicing potentials in CD44^+^ compared to CD44^−^ subpopulation (Fig. 4A & B).

### CD44^+^ cells were cisplatin-resistant

Freshly sorted CD44^+^, CD44^−^ and unsorted cells of H1299 and H1650 were cultured in serum-free RPMI medium and subjected to 5 µM cisplatin treatment for 24 hr. The histogram in Fig. 4C represented the average of three individual experiments for both cell lines. Apoptotic and non-viable cells were measured by Annexin V and PI staining using flow cytometry, respectively. For H1299, cisplatin-treatment of unsorted or CD44^+^ cells resulted in no significant increase of apoptotic cells compared with their untreated control, indicating relative cisplatin resistance. CD44^−^ cells showed a significant increase in apoptosis (***p<0.001) after treatment indicating cisplatin sensitivity. When comparing amongst the groups, CD44^−^ cells showed significant increases (### p<0.001) of apoptosis compared to both unsorted and CD44^+^ cells, indicating that CD44^−^ cells were the most cisplatin-sensitive of the 3 groups. Comparable results were also observed for H1650, indicating relative cisplatin resistance of the unsorted and CD44^+^ cells. Furthermore, resistant H1299 cells stably selected by long term cisplatin treatment showed a higher basal percentage (96.2%) of CD44^+^ cells compared to the parental cells (82%). Likewise, for H1650, basal CD44^+^ percentage increased from 61.5% to 92.9%. The results indicated resistance and a survival advantage of CD44^+^ cells under chronic cisplatin treatment (Supplementary Figure S2).

### Immunohistochemical analysis of cancer stem cell markers in NSCLC specimens

**Figure 5:**
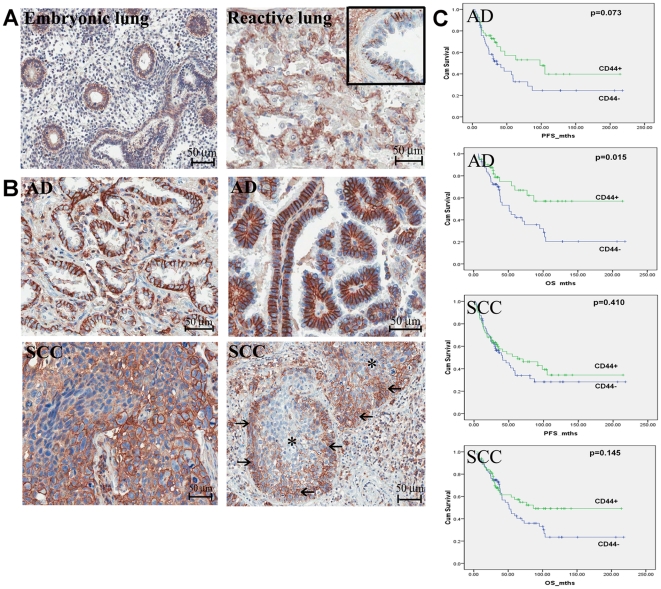
IHC analysis of human lung tissues. A. CD44 expression in pseudoglandular stage of embryonic lung (left) and regenerating pneumocytes of lung with diffuse alveolar injury (right). Inset shows CD44^+^ basal cells in normal bronchial epithelium. B. Representative cases of adenocarcinomas (AD) showing moderate (left) and strong (right) CD44 expression in cell membrane distribution. In some cases of squamous cell carcinomas (SCC), CD44 expression was particularly prominent in tumor cells at the periphery (arrow) than central regions (asterisks) of tumor islands which are generally regarded as stem cell sites. Scattered small lymphocytes and marcophages also showed CD44 expression in the tumor stroma. C. Kaplain Meier survival analysis of patients with AD (upper panel) and SCC (lower panel) stratified according to CD44^+^ (tumors with moderate/strong expression) or CD44^−^ (absent/weak, focal staining). PFS, progression-free survival in months; OS, ovrall survival in months.

To evaluate the *in vivo* protein expression of CD44 expression, IHC was performed on arrayed tumor cores of 141 primary lung carcinomas and reactive or fetal lung tissues. In the control tissues, cell membrane expression of CD44 was observed in basal cells of respiratory or metaplastic squamous bronchial epithelium, and regenerating cuboidal pneumocytes of injured lung. No expression was observed in terminally differentiated epithelial cells such as ciliated or non-ciliated columnar cells of bronchial epithelium, or type I flat pneumocytes lining alveolar spaces. In first trimester human fetal lung, CD44 was expressed in the epithelium of primitive airways (Fig. 5A). In lung cancers, expression was present in alveolar macrophages and small lymphocytes which served as internal positive controls. Totally, 62/141 (50.4%) cases showed CD44 expression. SCC was significantly associated with moderate to strong expression (21/27, 77.8%) compared to AD (41/96, 42.7%) (p = 0.002) (Fig 5B). In SCC showing moderate to well differentiation, CD44 was expressed much more strongly in the basal compared to maturing tumor cells (Fig. 5B), consistent with the expected stem cell niches. When only AD were considered, CD44 expression showed a weak association with non-smokers (p = 0.024). For AD, Log Rank test showed that patients with negative or weak CD44 expression had a significantly worse overall (p = 0.015) but not progression-free survival. For SCC, no significant relation of survival with the level of CD44 expression was observed. No association with other clinicopathological parameters including patient age, gender, tumor grade, size, stage, lymph node status, or pathological stage was observed (Supplementary Table S2). CD133 expression was detected only in scattered small cells in the stroma of fetal and reactive adult lung tissues. The basal bronchiolar epithelium, regenerating pneumocytes or tumor cells were negative. CD34 was detected in endothelium and reactive stromal cells in some tumors but not cancer cells. To ensure that the absence of staining was not due to regional variation of cellular distribution, the results were validated by repeating IHC analysis on full sections of cancer in selective cases.

## Discussion

The hypothesis that cancers are maintained by a subpopulation of stem or progenitor-like cells while non-stem/progenitor cells have a finite life span raises the possibility that targeting specific components of the regulatory pathways of cancer stem cell maintenance could provide a means of cancer control. As a preliminary step to investigate whether this hypothesis is applicable to lung carcinomas, it is necessary to identify and isolate cancer initiating cells using suitable markers. While the optimal approach for this purpose is still being explored, flow cytometric analysis and sorting of marker-positive cells is currently the most widely applied method [24]. Amongst the surface markers studied, we have shown that CD44^+^ cells are enriched for tumor propagating capacity and CD44 is a potential CSC marker of NSCLC cell lines. In a recent study on metastatic lung cancers in malignant pleural effusion, CD44 was also reported as a possible stem cell marker but the characterization of stem cell-like properties was based on *in vitro* molecular analysis and cellular expansion only [16]. In our study, *in vitro* and *in vivo* tumorigenecity of CD44^+^ cells was shown. Furthermore, our xenograft experiments showed a progressive enhancement of transplantation efficiency in successive tumor generations with shortening of latency period and decreased minimal cellular dose of engraftment. An increasing tumor formation capacity was also reported by Du et al. who investigated CD44^+^ subpopulations of colon cancer cells [25]. Breast epithelial cells induced to undergo EMT were shown to acquire stem cell-like and tumorigenic characters [26]. In our study, CD44^+^ cells were also associated with expression of markers implicating EMT such as *SNAI1*, *CDH2* and *VIM*. Only CD44^+^ cells expressed the pluripotency genes *POU5F1*, *NANOG* and *SOX2* [10], [27], [28], [29], while these markers were lost in CD44^−^ cells, suggesting that EMT may be involved in maintaining stemness. Further investigations are needed to elucidate the underlying mechanism and interaction between these transcription complex proteins and CD44 expression.

In cultured H1299 and H1650 cells, CD44^+^ cells demonstrated a lower basal apoptotic level and relative resistance to cisplatin treatment compared to CD44^−^ cells. This indicates their resilience against chemotoxicity and capacity to maintain tissue homeostasis, features believed to be associated with tissue stem cell phenotype. On the other hand, when CD44^+^ cells were transplanted from the *in vitro* to *in vivo* environment triggering marked cell loss through apoptosis, a 100 fold reduction of tumor cells was able to initiate tumors at a faster rate in sequential mice generations, demonstrating the robustness of the stem cell-like subpopulation contained in the selected cells. This could also indicate that only a portion of the CD44^+^ cells were required for tumor initiation, and that the increasing tumorigenecity of CD44^+^ cells was due to *in vivo* enrichment of CSC. Further marker studies and refined selection criteria are necessary for more specific *in vitro* CSC isolation.

CD133 is the most commonly reported marker and has been used to isolate CSC from fresh lung cancers [13], [15] but in our study, most cancer cell lines showed no significant CD133 expression by either semi-quantitative RT-PCR (data not shown), IHC, immunoblotting (Fig. 2A) or flow cytometry analysis (Table 1). Using the same anti-CD133 antibody, Chen et al. observed only 0.7% CD133 expression in H1299, while no expression was found in our study [11]. Amongst our studied cell lines, only HCC1833 showed SB formation on CD133-selection but *in vivo* tumorigenecity could not be demonstrated. Use of other mice species which are immunologically more tolerant towards xenografts might reveal different results. No data on HCC1833 are available in the literature for comparison. Stuelten et al also found infrequent CD133 expression in the NCI60 cancer cell panel [30]. CD44 expression was observed but in two cell lines, their findings differed from ours. We observed 0% and 95.9% of CD44 in A549 and H23, but 84.41% and 30.95%, respectively, were detected by the investigators [30]. We cannot explain the differences but data of other cancers have also reported discrepant observations. For example, one study reported 38–72% of CD133+ cells in the ovarian cancer cell line SKOV3 while the percentage found by Stuelten et al was only 0.78%. Different marker percentages have also been found in colon and liver cancer cells [30], [31], [32]. The inconsistency of CSC marker profile expression amongst different studies could be related to individual cancer variation, but they could also be due to different potency states, compositional or functional characteristics of the cancer stem or progenitor populations. In the absence of a specific marker, the true percentage of CSC in a tumor, particularly that in long-established cancer cell lines, is controversial [24]. The variation in environmental and selective pressures experienced by cancer cells *in vitro* and *in vivo* might trigger or suppress different pathways of the molecular networks that regulate CSC functions, and it is not clear whether the CSC marker profile could vary with circumstances. While we have demonstrated that CD44^−^ cells are incapable of tumor perpetuation, more refined methods for CSC selection and characterization are clearly needed. It would be worthwhile to explore whether CD44 could be combined with other potential CSC markers such as ALDH, CXCR4, ABCG2, side population marker, ESA, etc., for improving the efficiency and specificity of CSC selection [13], [21], [22], [33].

CD44 is a membrane bound glycoprotein which mediates a complex range of functions. Recent studies have provided support for its role as a CSC marker. For example, the clonal expansion and xenograft initiation capacity of CD44^+^-selected colorectal cancer CSC could be inhibited by CD44 knockdown [25]. Homozygous CD44 deletion affected intestinal crypt cell survival and attenuated tumorigenecity without affecting proliferation in a primary mouse colon carcinoma model [34]. Mechanistically, invasive and metastatic growth can be mediated through the interaction of cell surface CD44 with extracellular matrix components such as hyaluronan with subsequent changes induced in the cytoskeletal machinery of cancer cells [35]. CD44 expressed on the surface of colon cancer cells has been shown to facilitate binding to endothelial P- or L-selectin and increase tumor access to haematogenous spread [36]. CD44 is also networked to many signaling cascades that mediate tumor-enhancing functions. It acts as a co-receptor with neighboring EGFR or other ErbB family receptors tyrosine kinases [37], and can activate cell proliferation pathways indirectly through ligand presentation such as the scatter factor to its receptor c-MET [38]. Tumor cell survival could also be enhanced through activation of anti-apoptotic pathways such as the PI3K/AKT cascade [39], [40] and Bcl2 and Bcl-xL transcription factors [41]. A report on small cell lung cancers showed that activation of CD44-MAPK-PI3K signaling led to increased expression of uPA/uPAR and MDR1, resulting in enhanced invasive and multi-drug resistant cancer phenotypes [42]. It has been suggested that variant forms of CD44, especially CD44v6 which is transiently expressed during embryonic lung development, mediates tumor cell migration and invasion and can suffice for a CSC marker [43]. Notably, in our CD44^+^-derived SB and xenografts, mRNA of both the standard and variant forms of CD44 were expressed.

Immunohistochemical analysis using a monoclonal antibody against the standard form of CD44 showed that in non-cancer lung, CD44 is not expressed in terminally differentiated lung epithelium but is upregulated in sites generally regarded as reserve or stem cell niches and in regenerating alveolar lining cells of injured lungs. Our data as well as those from several studies have shown a significantly more frequent CD44 expression in squamous compared with adenocarcinoma histology. Interestingly, metaplastic squamous epithelium of bronchi displays increased hyaluronan and CD44 expression in the proliferating basal layers, while in premalignant dysplasia, the entire thickness shows aberrant hyaluronan-CD44 expression, indicating that squamous malignant transformation is closely associated with CD44 expression [44]. For AD, the picture is less clear, perhaps related to their heterogeneous histogenetic origin. In the literature, associations of immunohistochemical CD44 expression with either a better or a worse patient outcome have been reported [43], [45]. In our study, we have observed an association of CD44 expression with longer survival in AD (p = 0.015) (Fig. 5C). This observation is contrary to conventional expectation that tumors enriched for stem cell properties would be biologically more aggressive. On the other hand, *in vitro* models have shown that lower hyaluronan levels could promote angiogenesis and support tumor progression [39]. Given that CD44 is a receptor of hyaluronan, it would be worth investigating whether CD44 expression would also be reduced and associated with tumor progression and a worse patient outcome. In tumors showing extensive CD44 expression, it is possible that not all tumor cells are responsible for tumor initiation. Whether CD44 expression is regulated by non-stem cell related pathways in clinical tumors need to be examined. A better understanding of the role of CD44 and its interaction with hyaluronan and other binding partners in tumor initiation and progression would be required to clarify its relation with patient outcome.

In summary, this study has provided evidence that amongst reported CSC markers such as CD133, ALDH, CXCR4, ABCG2, ESA and side population staining, CD44 could be a potentially useful marker for lung cancers. Further experiments using more refined selection criteria such as a combination of two or multiple markers would be useful to specifically identify and purify CSC. Testing on resected tumor samples or pleural effusion fluid would help to clarify its applicability in clinical settings. Further investigations of the roles and mechanistic pathways of CD44 in tumor initiation and progression are required to validate its role in CSC maintenance and regulation, and provide further insight on its usefulness as a specific therapeutic target.

## Materials and Methods

### Cell lines and cultures

Ten human non-small cell lung cancer cell lines were obtained from ATCC or kindly provided by Dr. JD Minna. HKULC2 and HKULC4 cell lines were raised from Hong Kong patients as reported previously [46]. PLC8024 was a liver cancer cell line kindly provided by Dr. KW Chan [4]. All cells were maintained in RPMI medium with 10% fetal bovine serum supplement. Stable cisplatin-resistant H1299-CR and H1650-CR cell lines were generated by culturing the parental H1299 and H1650 cell lines, respectively, with increasing dose of cisplatin for 3 months. Single resistant clones were expanded in RPMI full medium and were retested to show cisplatin resistance.

### Animals

Ncr-nu/nu-nude mice were maintained under pathogen-free conditions. All animal experiments were performed according to National Institutes of Health guidelines and approved by the Institutional Animal Care and Use Committee at Nevada Cancer Institute (Animal protocol number 05-001).

### RNA isolation, cDNA synthesis and semi-quantitative reverse-transcription polymerase chain reaction

Total RNA was extracted using TRIZOL reagents (Invitrogen). One µg of the extracted RNA was used for cDNA synthesis using first-strand cDNA kit according to the manufacturer's instructions (Invitrogen) and subjected to semi-quantitative reverse-transcription polymerase chain reaction (RT-PCR). PCR amplifications for *POU5F1* (OCT4), *NANOG*, *SOX2*, *SNAI1*, *CDH2*, *VIM*, *PROM1* (CD133), *CD44s*, *CD44v3*, *CD44v5*, *CD44v6*, *CD44v10* and *GAPDH* were performed for 40 cycles using primers and conditions listed (Supplementary Table S1).

### Flow cytometry analysis and fluorescence-activated cell sorting

Expression of cancer stem cell markers was evaluated by flow cytometry. Dead cells, cell debris and doublets were first gated out using either PI or Live Dead dye (Invitrogen), or based on cell size and complexity. Single color compensation controls were performed in each experiment for non-specific spectral signals emitted by the fluorochromes [24], [47]. Cells were labeled with mouse origin direct fluorochrome-conjugated antibodies from BD Bioscience. The antibodies consistsed of anti-CD34-PE (Cat. No. 560941), anti-CD44-fluorescein isothiocyanate (FITC) specific for CD44 standard form (Cat. No. 555478), anti-OCT4-PE (Cat. No. 560186), anti-BMI1-FITC (Cat No. 612666)] and Miltenyi Biotech [anti-CD133-PE (Cat. No. 130-080-901)] at the concentration recommended by the manufacturers. For intracellular staining of OCT4 and BMI1, cells were prefixed and permeablized with Fix & Perm cell permeabilization Kit (Invitrogen) before adding the antibodies. Corresponding isotype-matched mouse immunoglobins were used as negative controls (BD Bioscience). Consistent labeling protocols were used in each experiment. At least 10,000 cells were acquired for each analysis. For cell sorting, labeling of cell surface markers was performed under sterilized conditions and cells were sorted by BD FACSVantage Cell sorter (BD Bioscience). The top 25% most brightly stained, and the lowest 20% most dimly-stained cells were selected as the positive and negative populations, respectively. Sorting purity of over 90% was ensured for further *in vitro* and *in vivo* experiments. All data were analyzed by the Flowjo software, version 5.7.2 (Tree star).

### Immunoblot

Cells were harvested and washed with 1× PBS and lysed on ice with RIPA Lysis buffer [10mM Tris, 150mM NaCl, 1mM ethylenediaminetetraacetic acid, 1% Triton X-100, 0.5% NP40, pH 7.4, freshly added 0.2mM PMSF in isopropanol, 1∶50 Phosphatase Inhibitor Cocktail 2 (Sigma), 1∶50 Protease Inhibitor Cocktail (Sigma)] for 1 hr. The cell lysate was then centrifuged at 13k rpm for 20 min at 4°C to remove cell debris. The protein amount in the lysate was quantified with the Dc Protein Assay (Bio-Rad). For each lysate, 30µg protein was loaded on SDS-PAGE and then transferred onto PVDF membranes (Amersham). The membranes were blocked by incubation with shaking in 1% BSA blocking buffer at room temperature for 1 hr. Primary antibodies of CD133 (Miltenyl Biotech), CD44 and ACTIN (Cell signaling) were diluted at 1∶1000 in TBS/Tween 20 with 5% BSA. Secondary antibody was diluted in 1% BSA blocking buffer. Target proteins on the membrane were visualized on X-ray films by using the ECL Plus Western Blotting Detection Reagents (Amersham, Buckinghamshire, UK). Results were obtained from at least 3 independent experiments and representative results were shown.

### Spheroid culture and *in vitro* serial transplantation

Freshly isolated CD44^+^ and CD44^−^ cells were cultured in low adherent 35mm dishes (Costar) under serum-free condition and supplemented with 20 µg/ml of insulin, 20 µg/ml EGF and 10 µg/ml of bFGF (Invitrogen) for 21 days according to published protocols [14]. In brief, SB were harvested every 3 days, centrifuged at 1200rpm for 5 mins and washed once with 1× PBS. SB were then re-cultured with fresh serum-free medium and supplements in low adherent dishes. Floating spheroid-like bodies (SB) were photographed by random field selection. Harvested SB were mechanically dissociated and seeded again in low adherent culture plates supplemented with growth factors for *in vitro* serial SB subculture. At least 3 passages were performed for each cell line. SB were also collected for immunofluorescence or RNA extraction and subsequent expression analyses. Data were collected from at least three independently performed experiments.

### Immunofluorescence staining for surface stem cell and ‘stemness’ gene markers

SB were stained with standard immunofluorescence (IF) protocol. Briefly, SB were put into 96 wells floating plate, and were stained with direct anti-CD44-FITC conjugated antibodies (BD Bioscience), or anti-OCT4, anti-NANOG and anti-SOX2 antibodies (Santa Cruz Biotechnology) at 4°C overnight. For intracellular staining of OCT4, NANOG and SOX2, cells were prefixed and permeablized with Fix & Perm cell permeabilization Kit (Invitrogen) according to manufacturer instruction before adding the antibodies. SB were then washed with 1× PBS for three times. Appropriate secondary antibodies (anti-rabbit-PE, anti-mouse-FITC or anti-goat-Texas Red) were added and images were visualized with a fluorescence microscope and imaged with a CCD camera.

### Apoptosis assay

For apoptosis assay, 5000 freshly isolated CD44^+^, CD44^−^ and unsorted cells were treated with vehicle or Cisplatin (5 µM) for 24 hr, cells were then cells were stained with Annexin V-FITC and PI according to the manufacturer's instruction (BD Bioscience). Cells were then washed and resuspended with 1× binding buffer. The numbers of Annexin V and PI-positive cells were counted by flow cytometer.

### Anchorage-independent growth assay

Unsorted, CD44^+/−^-sorted cells were suspended in soft agar and growth medium in 6-well plates at density of 5000 cells per well. After 3–4 weeks, colonies were counted under the microscope in 10 fields per well and photographed.

### *In vivo* tumorigenicity experiment and serial transplantation

For mouse xenografts, a range of numbers of freshly isolated unsorted, CD44^+^/^−^ and CD133^+/−^ from all seven cell lines were mixed with growth factors and matrigel, and injected (using 27-gauge needle) subcutaneously into the back of four weeks old nude mice (nu/nu). Mice were monitored for subcutaneous tumors weekly for up to 9 weeks. Tumor volume (TV) was calculated according to the formula: TV (cm^3^) = d^2^×D/2, where d and D were the shortest and the longest diameters, respectively. Tumor growth curves were plotted. When tumor diameters reached at least 1cm in size, mice were scarified and tumor tissues were collected for morphological assessement. All tumor tissues were harvested. Parts of the tumors were processed for morphological and immunohistochemical analysis. Remaining tissues were subjected to further flow cytometry marker analysis and *in vivo* serial transplant.

To test for *in vivo* serial transplantation, primary xenografts from H1299 CD44^+^ cells were digested with collagenase type II (2 mg/ml) under constant rotation for 2 hr at 37°C. Cells were washed, collected and passed through a 75 mm strainer (BD Bioscience). Cells were stained with anit-CD44-FITC antibodies, reanalyzed for CD44^+^ percentage and sorted. The unsorted, CD44^+^ and CD44^−^ cells were injected into secondary recipient nude mice and monitored for tumor formation. Subsequently, secondary tumors were sorted and transplanted into tertiary recipient mice by the same method. The required numbers of cells and latency period for tumor formation were recorded.

### CD44 and CD133 expression analysis in clinical tumors, xenograft tumors and cell blocks by IHC

Resected tumors of 141 primary NSCLC from untreated Chinese patients were studied for CD44 expression. Cores of tissues of 0.6mm diameter in 3 to 4 replicates were arrayed on paraffin blocks, using normal lung, liver and gastrointestinal tract tissues as control, and a constant sample of lung carcinoma for normalization across different tissue array blocks. Tumor typing, staging criteria and definitions of smoking history were as previously described [48]. Deparaffinized tissue sections and cell blocks were blocked by background sniper (BiocareMedical) and peroxidase (BiocareMedical). Sections were labeled with primary antibodies against CD34 (clone QBEnd/10; BiocareMedical) and CD44 standard form (clone 156-3C11; BiocareMedical) and CD133 (clone CD133/1; Mitenyl Biotech) at 1∶200 dilution for 30 mins at room temperature. Subsequent steps of polymer-based detection system were performed according to the manufacturer's suggestions (BiocareMedical). For xenograft tumor, the same primary antibodies were used but sections were treated with biotin-blocking (DAKO) and mouse Ig blocking reagents. The Mouse on Mouse Immunodetection kit (Vector Laboratories) comprising IgG biotinylated anti-mouse secondary antibodies and ABC reagents (Vector Laboratories) were used and color detection was performed by DAB substrates (BiocareMedical). For control, the primary antibody was replaced with universal negative control serum (BiocareMedical). Images of stained sections were acquired by an automated scanner and the protein expression level was semi-quantitatively measured by an image analysis system (Aperio). Tumor cells not showing any degree of staining were counted as negative. Those showing only faint staining were considered weak; cells showing membrane staining similar in intensity to basal cells of bronchiolar epithelium were considered moderate, and those with more intense staining were considered as strong. Tumors showing any proportion of cells with moderate or strong staining were considered as positive. Tumors showing none or weak staining were grouped and compared to those showing positive expression.

### Statistical analysis

Differences between experimental groups were analyzed by One way ANOVA (Bonferroni multiple group analysis test) for SB formation, clonogenicity and cisplatin sensitivity study using GraphPad PRISM software, version 3.0 (GraphPad Prism). Statistical analysis of IHC results and comparison with clinicopathological data were performed by the χ2 test, Fisher exact test or Mann-Whitney U test where appropriate, and survival data by Kaplan Meier analysis using SPSS version 16.0 (SPSS Inc.). The two-sided significance level was set at p<0.05.

## Supporting Information

Figure S1 Percentage of Spheroid Bodies (SB) Formation of Unsorted and Sorted Cells in Seven NSCLC Cell Lines.
(10.38 MB TIF)

Figure S2 Representative Flow Cytometry Diagrams of Basal CD44 Percentage Analysis of Parental and Cisplatin-resistant H1299 and H1650 Cell Lines.
(8.09 MB TIF)

Table S1 PCR Conditions and Primer Sequences.
(0.03 MB XLS)

Table S2 Immunohistochemical CD44 Expression and Clinicopathological Profile of NSCLC.
(0.03 MB XLS)
