# Supplementary figures and images for: Non-Small Cell Lung Cancer Cells Expressing CD44 Are Enriched for Stem Cell-Like Properties
Source: PLoS One. 2010 Nov 19;5(11):e14062. doi: 10.1371/journal.pone.0014062 (PMC2988826; doi:10.1371/journal.pone.0014062)

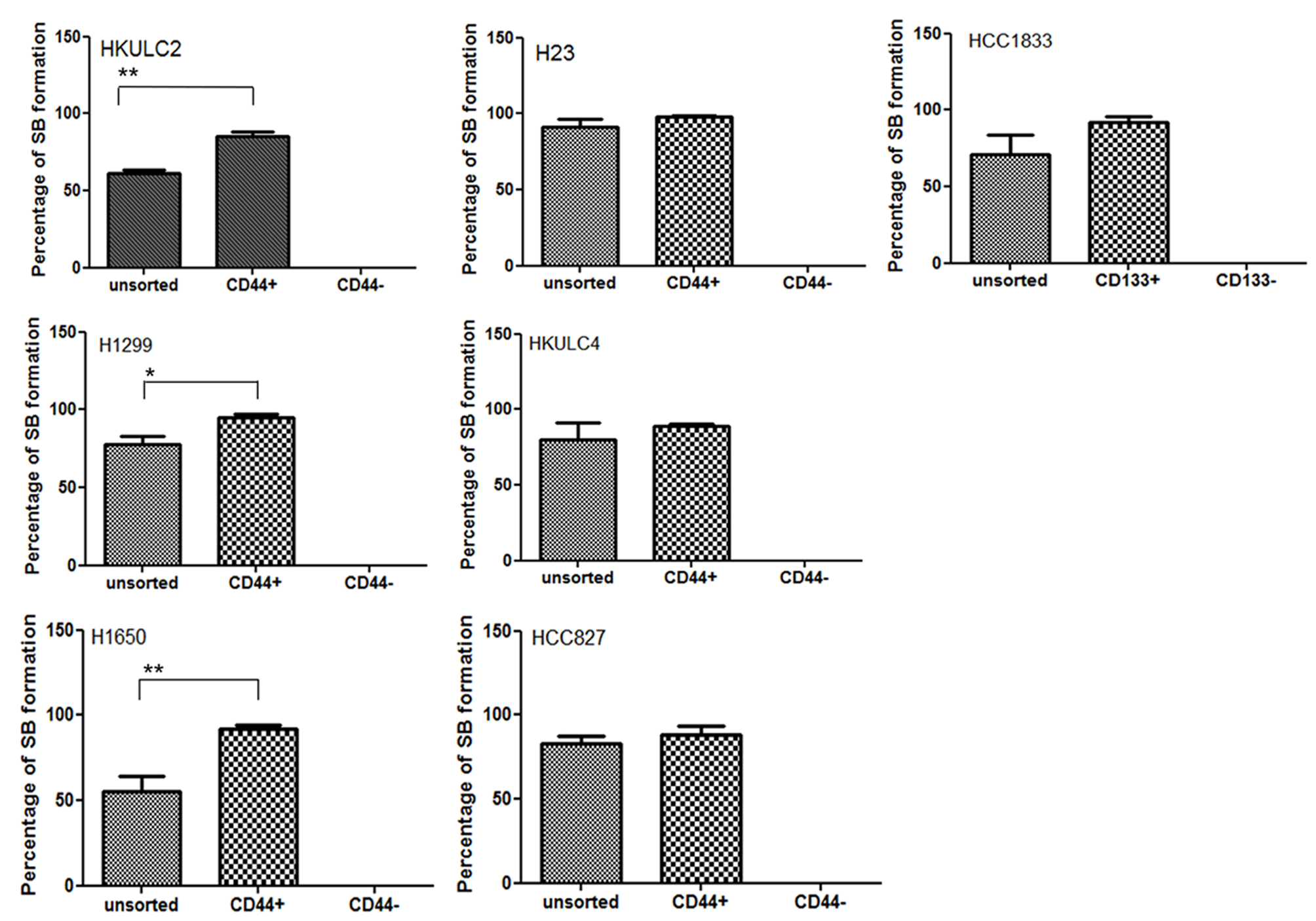

Supplement: Figure S1 — Percentage of Spheroid Bodies (SB) Formation of Unsorted and Sorted Cells in Seven NSCLC Cell Lines. (10.38 MB TIF) [file pone.0014062.s001.tif]

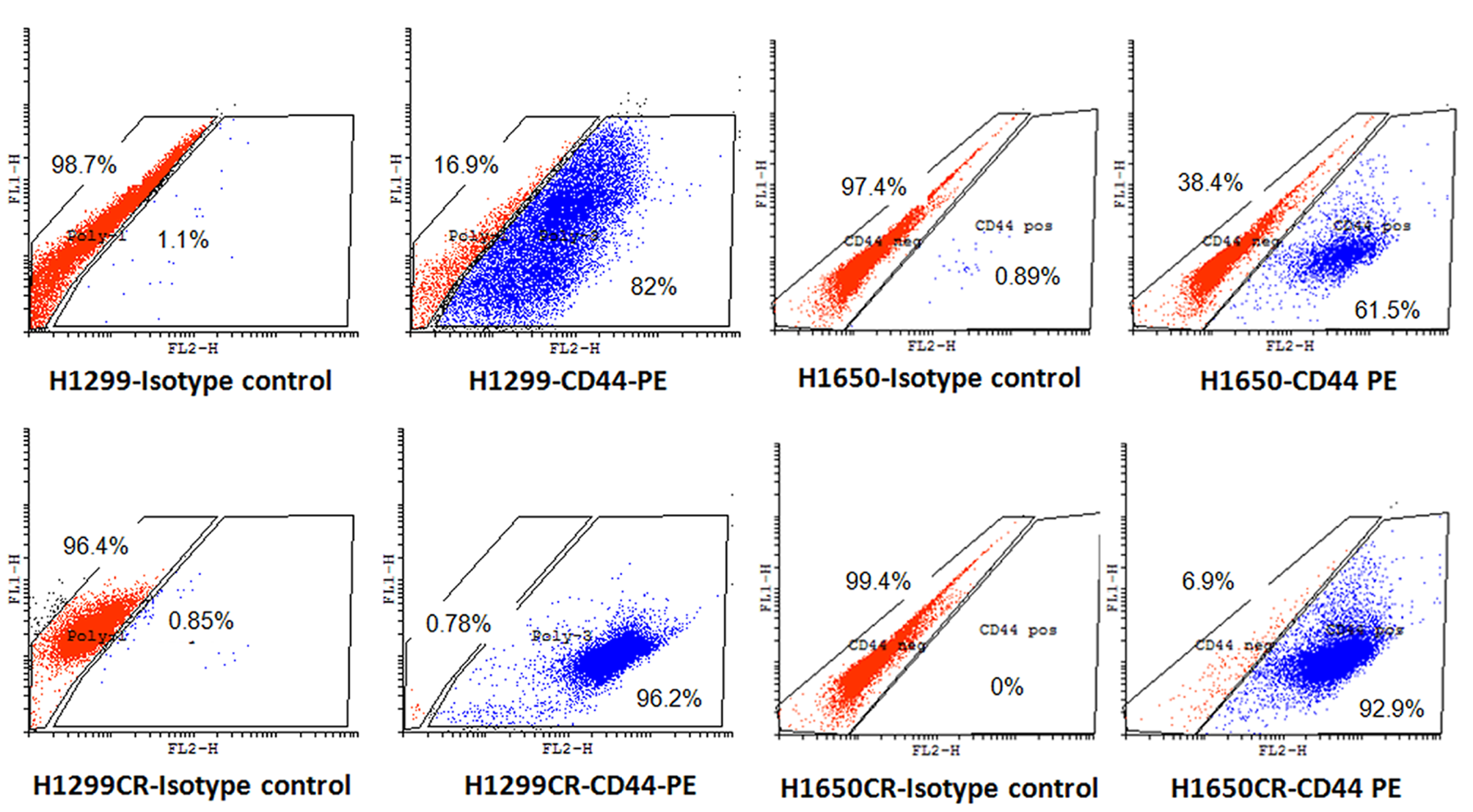

Supplement: Figure S2 — Representative Flow Cytometry Diagrams of Basal CD44 Percentage Analysis of Parental and Cisplatin-resistant H1299 and H1650 Cell Lines. (8.09 MB TIF) [file pone.0014062.s002.tif]
